# Spatiotemporal Properties of the Action Potential Propagation in the Mouse Visual Cortical Slice Analyzed by Calcium Imaging

**DOI:** 10.1371/journal.pone.0013738

**Published:** 2010-10-29

**Authors:** Makoto Osanai, Satoshi Tanaka, Yusuke Takeno, Shouta Takimoto, Tetsuya Yagi

**Affiliations:** Division of Electrical, Electronic and Information Engineering, Graduate School of Engineering, Osaka University, Suita, Japan; Vrije Universiteit Amsterdam, Netherlands

## Abstract

The calcium ion (Ca^2+^) is an important messenger for signal transduction, and the intracellular Ca^2+^ concentration ([Ca^2+^]_i_) changes in response to an excitation of the cell. To reveal the spatiotemporal properties of the propagation of an excitatory signal with action potentials in the primary visual cortical circuit, we conducted a Ca^2+^ imaging study on slices of the mouse visual cortex. Electrical stimulation of layer 4 evoked [Ca^2+^]_i_ transients around the stimulus electrode. Subsequently, the high [Ca^2+^]_i_ region mainly propagated perpendicular to the cortical layer (vertical propagation), with horizontal propagation being restricted. When the excitatory synaptic transmission was blocked, only weak and concentric [Ca^2+^]_i_ transients were observed. When the action potential was blocked, the [Ca^2+^]_i_ transients disappeared almost completely. These results suggested that the action potential contributed to the induction of the [Ca^2+^]_i_ transients, and that excitatory synaptic connections were involved in the propagation of the high [Ca^2+^]_i_ region in the primary visual cortical circuit. To elucidate the involvement of inhibitory synaptic connections in signal propagation in the primary visual cortex, the GABA_A_ receptor inhibitor bicuculline was applied. In this case, the evoked signal propagated from layer 4 to the entire field of view, and the prolonged [Ca^2+^]_i_ transients were observed compared with the control condition. Our results suggest that excitatory neurons are widely connected to each other over the entire primary visual cortex with recurrent synapses, and inhibitory neurons play a fundamental role in the organization of functional sub-networks by restricting the propagation of excitation signals.

## Introduction

A fundamental part of neuroscience is the characterization of neuronal circuits. The calcium ion (Ca^2+^) is an important messenger for signal transduction, and the intracellular Ca^2+^ concentration ([Ca^2+^]_i_) has been shown to change in response to the excitation of the cell [1]–[5]. Thus, an analysis of [Ca^2+^]_i_ dynamics may help to better characterize the behavior of neuronal circuits.

Often, extracellular recordings have been conducted using a single microelectrode to reveal the signal propagation properties in the visual cortex [6]–[10]. However, understanding the behavior of a neuronal circuit based on single-site neuronal recordings is difficult. To understand signal processing in neuronal networks better, the activity of a large population of neurons should be simultaneously measured. As a direct approach to reveal circuit dynamics, multi-electrode recordings have been conducted [11]–[13]. A multi-electrode array can record the spikes or local field potentials from an ensemble of neurons simultaneously. Unfortunately, this approach is also not optimal, because it has the disadvantage of sampling only a small population of neurons [1], [2].

The spatiotemporal properties of visual cortical signal propagation have been investigated using voltage-sensitive dyes [14]–[20]. The major signal source for such dyes is thought to be postsynaptic potentials rather than action potentials [21]. In neurons, an action potential opens voltage-dependent Ca^2+^ channels and causes [Ca^2+^]_i_ to increase in the cell somata [1]–[3]. Therefore, the propagation of excitatory signals that induce super-threshold activations in circuits can be discriminated using the Ca^2+^ imaging technique. Another merit of the Ca^2+^ imaging technique is the better signal-to-noise ratio when compared with voltage-sensitive dye imaging [1], [2].

Ca^2+^ is also involved in signal transduction in the cell and the modulation of protein functions (e.g., enzymes, ion channels, and receptors). Due to these functions, Ca^2+^ can affect synaptic transmissions, gene expression, and morphological changes of cellular processes [4], [5], [22]. In the nervous system, these modulations and/or changes of cellular properties are the basis for changes of neuronal properties (i.e., synaptic plasticity) [23], [24] (for a review, see Ref. [25], [26]). Therefore, the measurement of the [Ca^2+^]_i_ transient allows the detection of the excitability of a neuron and helps to reveal the plastic changes of the synaptic transmission.

Here, we demonstrate the spatiotemporal properties of the [Ca^2+^]_i_ changes evoked by layer 4 stimulation in primary visual cortical slice preparations by means of Ca^2+^ imaging to study the propagation of action potentials in the primary visual cortical neuronal networks. These results also provide quantitative data to study the plasticity of the neuronal circuits, which is highly dependent to [Ca^2+^]_i_ alterations.

Some of our preliminary results have been previously reported at meetings [27]–[29].

## Results

### Spatiotemporal signal propagation properties

**Figure 1:**
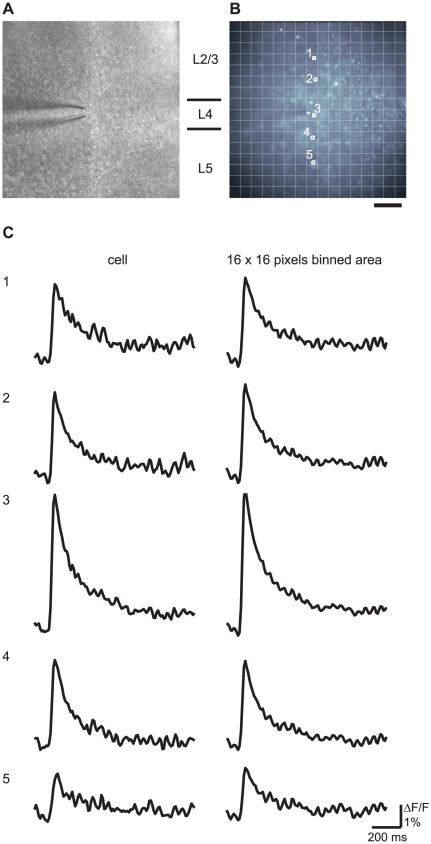
[Ca^2+^]_i_ transients induced by the electrical stimulation of layer 4 of the visual cortex. (A) IR-DIC image of the visual cortical slice and stimulus electrode. (B) Fluorescent image of the visual cortical slice loaded with OGB1-AM. The tip of the stimulus electrode is indicated by an asterisk (*). The number and approximate borders of the cortical laminae between (A) and (B) are indicated. Scale bar = 100 µm. (C) Time courses of the [Ca^2+^]_i_ transients evoked by the application of an 80 µA stimulus in layer 4 obtained from the cell soma region shown in the upper panel (left column) and from the 16×16-pixel binned area including the cell soma region (right column). The numbers correspond to the cell number shown in the image in (B). Scale bar = 200 ms; ΔF/F = 1%.

Figure 1 depicts the fluorescent photomicrographs (Figure 1B) and typical time courses of the [Ca^2+^]_i_ transients induced by electrical stimulation at layer 4 in the cell body regions in each of the cortical layers (Figure 1C, left column) and in the 16×16-pixel binned area containing the cell bodies (Figure 1C, right column). The IR-DIC image of the visual cortical slice and the stimulus electrode is shown in Figure 1A. Transient [Ca^2+^]_i_ elevation was observed after stimulation of the visual cortical neurons. The [Ca^2+^]_i_ transient with the largest amplitude was observed in layer 4 cells, and the [Ca^2+^]_i_ transients from layer 2/3 neurons were larger than those from layer 5 neurons. The latencies of the [Ca^2+^]_i_ transients were within 10 ms (less than one frame) near the stimulus position and within 20 ms in the other regions. The time-to-peak values were 40–50 ms in each neuron. The time courses of the [Ca^2+^]_i_ transients obtained from the cell body region and from the binned area were nearly identical. This observation indicates that the fluorescent signal in the cell body region may actually include out-of-focus areas just above and below the cells. To avoid the incorporation of any misleading information, 16×16-pixel binned data were used for quantitative data analyses (see Discussion).

**Figure 2:**
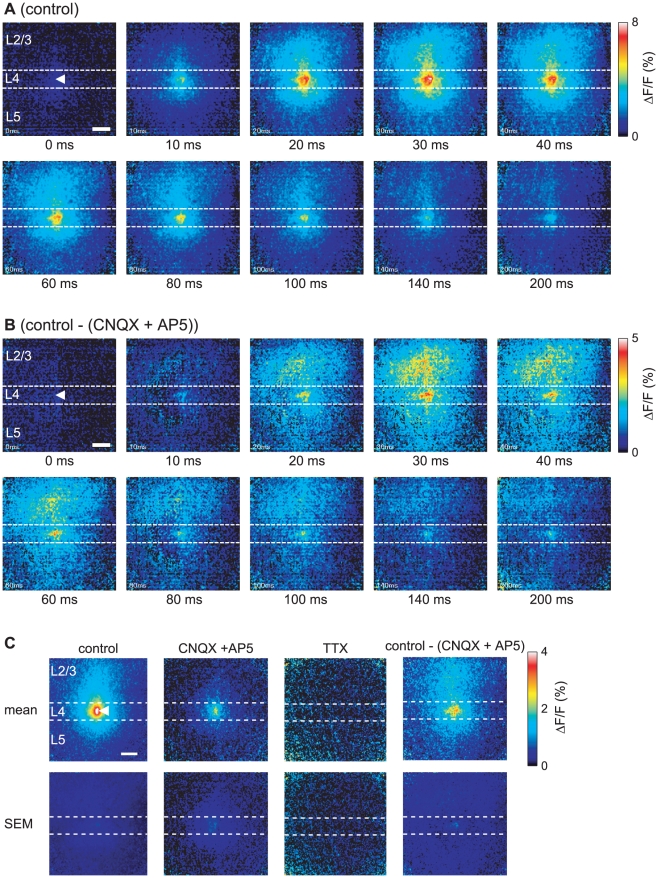
Spatiotemporal properties of signal propagation evoked by layer 4 stimulation. Time-lapse pseudocolor images of the [Ca^2+^]_i_ transients evoked by application of an 80 µA stimulus in layer 4 (white arrowhead) under the control condition (A) and of the orthodromic response obtained by subtraction of the ΔF/F values under the administration of CNQX and AP5 from those under the control condition (B). The time elapsed after stimulation is shown under each image. (C) Distributions of mean values (mean) and errors (SEM) of the peak amplitudes of the [Ca^2+^]_i_ transients evoked by 80 µA layer 4 stimulation under the control condition (control, n = 9 slices), the administration of CNQX and AP5 (CNQX + AP5, n = 5 slices), and the administration of TTX (TTX, n = 4 slices), as well as of the mean and error values obtained by subtraction of the ΔF/F values under the administration of CNQX and AP5 from those under the control condition (control − (CNQX + AP5), n = 5 slices). Scale bar = 100 µm.

Figure 2 depicts the pseudocolor time-lapse images converted from the ΔF/F values in each pixel from layer 4 stimulation (Figure 2A, B) and the spatial distribution of the maximum amplitudes of the [Ca^2+^]_i_ transients (Figure 2C). The [Ca^2+^]_i_ transients were initiated around the stimulus position within 10 ms and increased in size up to 30 ms after stimulation. The high [Ca^2+^]_i_ region spread vertically toward layer 2/3 over the observed region (approximately 300 µm) 20–30 ms after stimulation, and the [Ca^2+^]_i_ transients in layer 2/3 increased in size up to 40 ms after stimulation. In contrast, the horizontal spread of the high [Ca^2+^]_i_ region was restricted to approximately 200 µm in layers 4 and 2/3. The high [Ca^2+^]_i_ region gradually shrank, starting approximately 60 ms after stimulation, and the [Ca^2+^]_i_ level returned to a near-basal level approximately 200 ms after stimulation. When the 80 µA stimulus was applied, the width of a high [Ca^2+^]_i_ region, in which the amplitude of [Ca^2+^]_i_ transient exceeded 30% of that observed at the stimulus position under the control condition, were 204±29 µm, 125±18 µm, 107±16 µm, and 109±14 µm in the dorsal, ventral, lateral, and medial directions, respectively (n = 9 slices; Figure 2C control). The response widths in the dorsal direction were significantly different from those in the lateral and medial directions (*p*<0.01; Friedman test with Tukey's post hoc test). Blocking the excitatory synaptic transmission by the application of CNQX and AP5 reduced the [Ca^2+^]_i_ transients around the stimulus position, (Figure 2C, CNQX+AP5). Under this condition, 80 µA stimulation caused response widths in the dorsal, ventral, lateral, and medial directions from the stimulus position of 36±16 µm, 33±17 µm, 35±18 µm, and 29±15 µm, respectively (n = 5 slices; Figure 2C CNQX+AP5), all of which were significantly smaller than those under the control condition (*p*<0.05; unpaired *t*-test). Anisotropic signal propagation could not be detected (i.e., there were no significant differences among the response widths in all directions; *p*>0.05; Friedman test with Tukey's post hoc test). No [Ca^2+^]_i_ transients were observed after TTX administration (n = 4 slices; Figure 2C TTX). To reveal the region of orthodromic signal propagation (postsynaptic response), [Ca^2+^]_i_ transients under the administration of CNQX and AP5 were subtracted from those under the control condition, pixel-by-pixel (Figure 2B and C control - (CNQX+AP5)). A patchy cluster of high [Ca^2+^]_i_ region can be seen above the stimulation site in layer 2/3, indicating where neurons might receive synaptic inputs from the neurons activated by the stimulation. These patchy structures likely correspond to the cell bodies (Figure S2; see discussion). The horizontal width of the high [Ca^2+^]_i_ region was wider in layer 2/3 than in layer 4.

**Figure 3:**
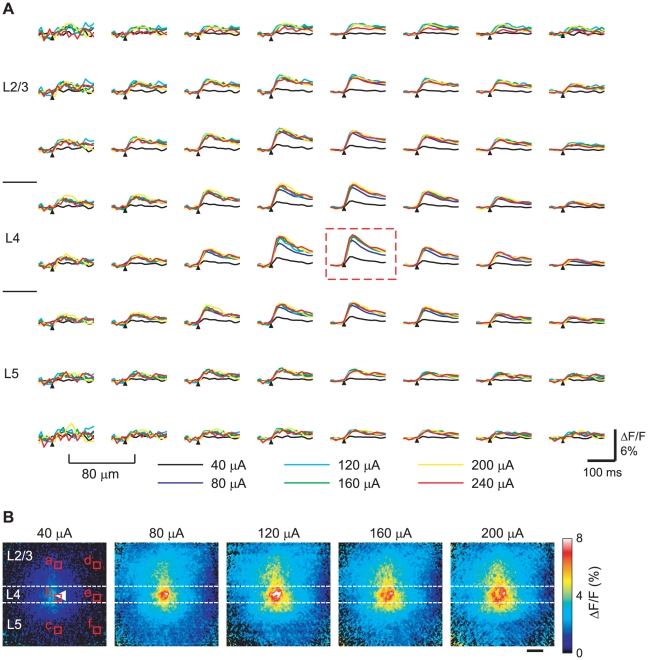
Typical time courses and spatial distributions of the [Ca^2+^]_i_ transients evoked by layer 4 stimulation. (A) Time courses of the evoked [Ca^2+^]_i_ transients obtained from the 16×16-pixel binned area with various stimulus intensities (40–240 µA). The slice is the same as that shown in Figure 2. The 8×8 time courses of the evoked [Ca^2+^]_i_ transients obtained from every alternate 16×16-pixel binned area are shown. The region where the stimulus was applied is indicated by a red dashed-line box. The center-to-center distance of each panel was 80 µm. Scale bar = 100 ms; ΔF/F = 6%. (B) Distributions of the peak amplitudes of the [Ca^2+^]_i_ transients evoked by various stimulus intensities. The slice is the same as that shown in (A). Pseudocolor images were used to show the maximum values of ΔF/F from each pixel. The stimulus intensities are given at the top of each image. The stimulus position is shown by an arrowhead in the leftmost image. Scale bar = 100 µm.

The typical time courses of the [Ca^2+^]_i_ transients obtained from the binned regions from the same slice shown in Figure 2 are shown in Figure 3A. In this slice, prominent [Ca^2+^]_i_ transients were observed only after the application of >80 µA stimuli. The application of >80 µA stimuli produced amplitudes and time courses for the [Ca^2+^]_i_ transients that were almost identical. Only small [Ca^2+^]_i_ transients were observed at the lateral and medial edge regions, even after application of a 240 µA stimulus. There were slight differences in the latencies and time-to-peaks among various stimulus intensities. The spatial distribution of the [Ca^2+^]_i_ transients at various stimulus intensities are shown in Figure 3B. When >80 µA stimuli were applied, dorsal elongation of the [Ca^2+^]_i_ elevation was observed. The time courses of the [Ca^2+^]_i_ transients in the same slice as shown in Figures 2 and 3 under the control condition or after blocking excitatory synaptic transmissions or action potentials are shown in Figure S3.

**Figure 4:**
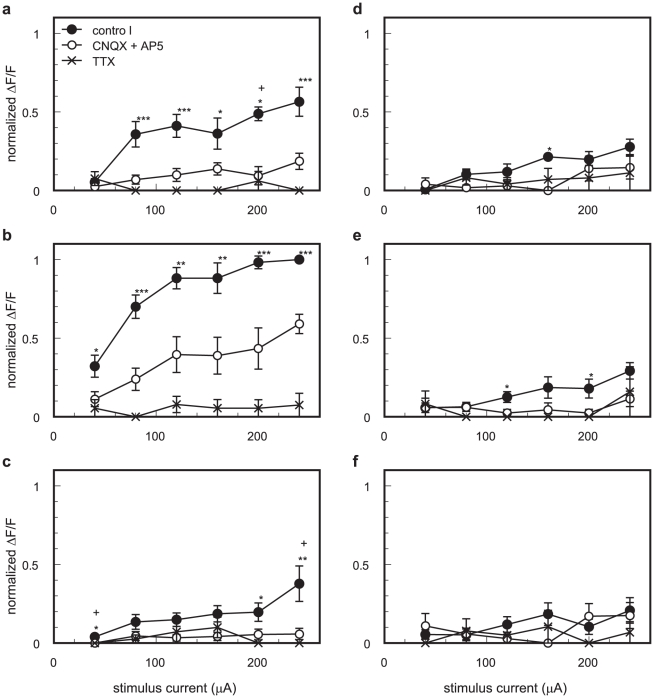
Intensity-response curves of the [Ca^2+^]_i_ transients. The relationships between the amplitude of the evoked [Ca^2+^]_i_ transients and the stimulus intensity in each of the regions under the control condition (solid circle), under the condition of 10 µM CNQX and 50 µM AP5 administration (open circle), and under the condition of 1 µM TTX administration (cross) are shown. The normalized ΔF/F values were obtained from the maximum amplitudes of ΔF/F from the 16×16-pixel binned area indicated in the leftmost panel of Figure 3B (red square) and were normalized by the amplitude evoked by application of the 240 µA stimulus at the stimulus position (region b) under the control condition. The values obtained from each slice were averaged (*n* = 5, 5, and 4 slices for the control, CNQX + AP5, and TTX conditions, respectively). The alphabetical index of each panel corresponds to the region indicated in leftmost panel of Figure 3B. The asterisks (*) or plus signs (+) indicate that the amplitude was significantly different from the amplitude under the conditions of TTX or CNQX and AP5 administration, respectively. Error bars are represented as SEM. *, +: *p*<0.05, **: *p*<0.01, ***: *p*<0.005, Kruskal-Wallis test with Dunn post hoc test.

The relationship between the stimulus strength and the [Ca^2+^]_i_ transients in the regions indicated in the left panel of Figure 3B are shown in Figure 4. Under the control condition, at the stimulus position (Figure 4b) and at 200 µm dorsally away from the stimulus position (layer 2/3, Figure 4a), the responses increased as the stimulus strength increased but plateaued with the application of >120 µA stimuli. The responses from other regions (Figures 4c, d, e, and f) were unremarkable regardless of the stimulus intensities, with the only exception being the response at 200 µm ventrally away from the stimulus position (layer 5, Figure 4c) under a 240 µA stimulus. Under the administration of CNQX and AP5, remaining responses were observed only near the stimulus position (Figures 2C, 4, and S3) even when large electrical stimuli were applied. CNQX and AP5 administration reduced the amplitude of the [Ca^2+^]_i_ transients at the stimulus position (region b of Figure 3B) to 35.1±9.6% and 42.7±12.8% of the control condition in the cases of the 80 and 200 µA stimuli, respectively (n = 5 slices). The amplitudes were significantly smaller compared with those of the control condition (*p*<0.005 and *p*<0.02, respectively; one-sample *t*-test). CNQX and AP5 also reduced the amplitudes at layer 2/3 (region a of Figure 3B) to 23.8±11.7% and 17.8±10.9% of the control condition in the cases of the 80 and 200 µA stimuli, respectively (*n* = 5 slices; *p*<0.005 and *p*<0.002, respectively; one-sample *t*-test). These results, combined with the results of the response widths, suggested that excitatory synaptic transmission contributed to the propagation of the high [Ca^2+^]_i_ region (see Discussion). TTX almost completely blocked the responses in all regions regardless of the stimulus intensities (n = 4 slices; Figure 4). The amplitudes of the [Ca^2+^]_i_ transients at the stimulus position (region b of Figure 3B) and at layer 2/3 (region a of Figure 3B) were 0.0±0.0% and 0.0±0.0% of the control condition in the case of the 80 µA stimuli (n = 4 slices; *p*<0.0001; one-sample *t*-test), and 4.9±4.9% and 10.6±10.6% in the case of the 200 µA stimuli (n = 4 slices; *p*<0.001 and *p*<0.005, respectively; one-sample *t*-test; note: only one slice exhibited small responses in the case of 200 µA stimulation). These results indicated that action potentials involved in the induction of the [Ca^2+^]_i_ transients (see Discussion).

### Effect of the blockade of inhibitory synaptic transmission

**Figure 5:**
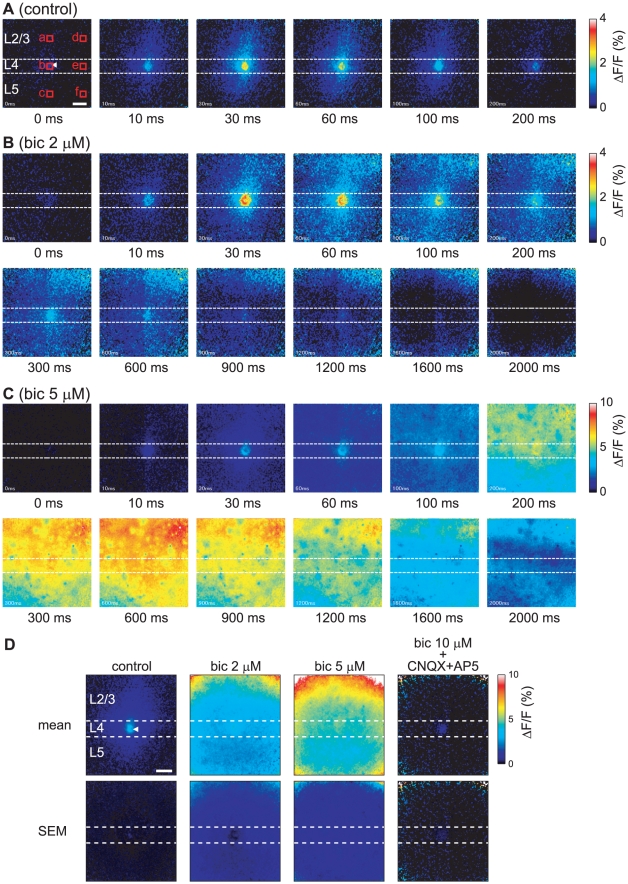
Blockade of inhibitory synaptic transmission enhanced the evoked signal propagation. Time-lapse pseudocolor images of the [Ca^2+^]_i_ transients evoked by the application of an 80 µA stimulus in layer 4 under the control condition (A), under the condition of 2 µM bicuculline administration (B), and under the condition of 5 µM bicuculline administration (C). The illustrations are the same as those in Figure 2. (D) Distributions of mean values (mean) and errors (SEM) of the peak amplitudes of the [Ca^2+^]_i_ transients evoked by 80 µA layer 4 stimulation under the control condition, under the administration of 2 and 5 µM bicuculline (n = 4 slices), and under administration of 10 µM bicuculline with 10 µM CNQX and 50 µM AP5 (n = 3 slices) are shown. Scale bar = 100 µm.

As mentioned above, layer 4 stimulation evoked the [Ca^2+^]_i_ transients, and the evoked signal propagated primarily in a dorsal direction toward layer 2/3, with horizontal signal propagation being restricted. To elucidate the mechanism behind this horizontal restriction, we investigated the signal propagation properties under a blockade of inhibitory synaptic transmissions. Figures 5 and S4 show the time-lapse images of the [Ca^2+^]_i_ transients and the time courses of the [Ca^2+^]_i_ transients, respectively, that are evoked by layer 4 stimulation under the control condition (Figures 5A and S4, black line), after the application of 2 µM (Figures 5B and S4, red line), and after the application of 5 µM bicuculline (Figures 5C and S4, blue line) in the same slice. The average spatial distribution pattern of the [Ca^2+^]_i_ transients under the administration of various concentrations of bicuculline is shown in Figure 5D. Under the administration of 2 µM bicuculline, the latency and initial slope of the [Ca^2+^]_i_ transients were almost identical to those under the control condition (Figure S4). The amplitudes of the [Ca^2+^]_i_ transients were slightly increased by the application of 2 µM bicuculline, and the decay times were slowed, particularly near the stimulus position and in layer 2/3. When 5 µM bicuculline was applied, a [Ca^2+^]_i_ transient was initiated near the stimulus position immediately after the stimulation, and weak and broad [Ca^2+^]_i_ elevations were observed (Figure 5C, 30–60 ms). Approximately 200 ms after stimulation, the largest [Ca^2+^]_i_ transients were observed in layer 2/3, followed by the propagation of the high [Ca^2+^]_i_ region ventrally toward layer 5 (200–1200 ms after stimulation). There was no horizontal restriction of signal propagation under the administration of 5 µM bicuculline, and the evoked signal propagated over the entire observation area.

The onset of the [Ca^2+^]_i_ transients did not differ between the control conditions and those under the administration of 5 µM bicuculline (Figures 5 and S4). Treatment with 5 µM bicuculline, however, slowed down the time-to-peak of the [Ca^2+^]_i_ transients over the entire observation area (Figures 5, S4, and S5). The time-to-peak value at the stimulus position under the control condition and the 5 µM bicuculline condition were 33±3 ms (*n* = 9 slices) and 415±113 ms (*n* = 4 slices), respectively (*p*<0.05, unpaired *t*-test) (Figure S5).

**Figure 6:**
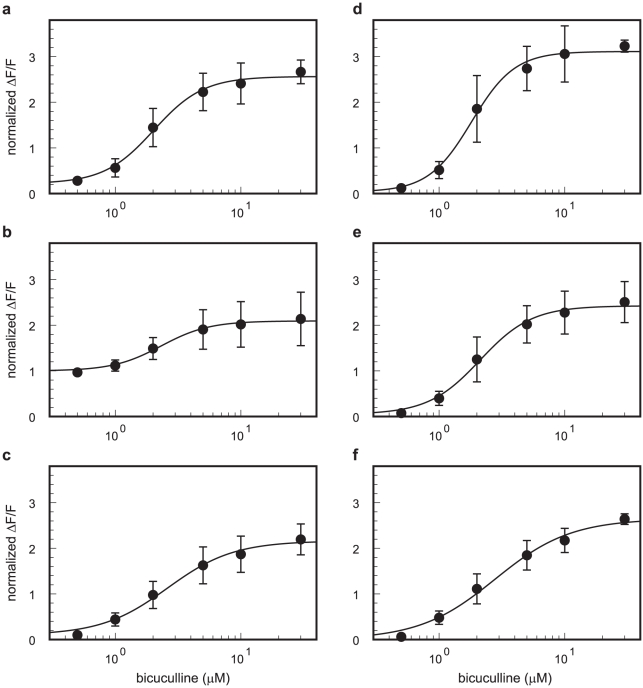
Effects of bicuculline application on the evoked [Ca^2+^]_i_ transients. Dose-response curves of the evoked [Ca^2+^]_i_ transients to bicuculline administration in each of the regions. The normalized ΔF/F values (solid circle) were obtained from the maximum amplitudes of ΔF/F from the 16×16-pixel binned area indicated in the leftmost panel of Figure 5A (red square). The ΔF/F values were normalized by the amplitude in the stimulus position (region b) under the control condition. The values obtained from each slice were averaged (*n* = 4 slices). The stimulus intensity was 80 µA. The alphabetical index of each panel corresponds to the region indicated in leftmost panel of Figure 5A. The normalized ΔF/F values were fitted into the following equation (solid line): *Base* + *A_max_*/(1*+* (*EC_50_*/[*bicuculline*])*^nH^*). The fitted *A_max_*, *EC_50_*, and *nH* values were as follows: region a, *A_max_* = 2.4, *EC_50_* = 2.0 µM, and *nH* = 2.2; region b, *A_max_* = 1.1, *EC_50_* = 2.3 µM, and *nH* = 2.3; region c, *A_max_* = 2.1, *EC_50_* = 2.6 µM, and *nH* = 1.6; region d, *A_max_* = 3.1, *EC_50_* = 1.8 µM, and *nH* = 2.5; region e, *A_max_* = 2.4, *EC_50_* = 2.1 µM, and *nH* = 2.1; and region f, *A_max_* = 2.6, *EC_50_* = 2.8 µM, and *nH* = 1.4. Error bars are represented as SEM.

The dose-response curves of the evoked [Ca^2+^]_i_ transients obtained from the peak amplitudes of the 16×16-pixel binned ΔF/F data in the regions indicated in Figure 5A (leftmost panel) are shown in Figure 6. The amplitude of the [Ca^2+^]_i_ transients increased in a dose-dependent manner. In particular, the signal evoked in layer 4 under the administration of 5 and 10 µM bicuculline propagated over the entire observation area (Figures 5 and 6). As shown in Figure 6, the effect of bicuculline on the [Ca^2+^]_i_ transients is described by Hill's equation, and the largest effect of bicuculline was observed in layer 2/3. These [Ca^2+^]_i_ transients almost completely disappeared under the administration of CNQX and AP5 with bicuculline (Figure 5D, rightmost panel). Together, these data suggest that signal propagation is due to excitatory synaptic transmission, and the blockade of inhibitory synapses enhances the propagation of the excitatory signals in the visual cortical circuit, particularly in layer 2/3.

Bicuculline has also been reported to block small-conductance calcium-activated potassium channels (SK channels) [33]. Blocking SK channels may diminish slow after-hyperpolarization and may raise the excitability of a neuron. To exclude any potential side effects of bicuculline, picrotoxin (n = 4 slices), another blocker of GABA_A_ receptors that does not block the SK channels, was applied to the slices. The typical time-lapse images of the [Ca^2+^]_i_ transients evoked by layer 4 stimulation under the control condition and after the application of 10 µM picrotoxin in the same slice are shown in Figure S6. The spatiotemporal properties of the signal propagation under the administration of 10 µM picrotoxin resembled those under the administration of 5 µM bicuculline, despite some differences in the amplitudes near the horizontal edges of the measurement area. In addition, Khawaled et al. [33] reported that at concentrations of <5 µM, the free base of bicuculline ((+)-bicuculline) has no prominent effect on SK channel currents (Figure 6 in Ref. [33]). Therefore, bicuculline should mainly have affected the GABA_A_ receptors directly in our study.

GABA_B_ receptors are known to be involved in slow inhibitory postsynaptic potentials (slow IPSPs) [34], [35]. The contribution of the GABA_B_ receptors to signal propagation in the visual cortex was also tested by the application of 1–30 µM CGP55845, a GABA_B_ receptor antagonist. No significant changes in the properties of the signal propagation were observed (n = 3; data not shown). These results strongly suggest that synaptic inhibition using GABA_A_ receptors plays a crucial role in confining the action potentials within a functional sub-network in the visual cortical circuit.

## Discussion

### Physiological implications of the [Ca^2+^]_i_ transients

In this paper, we demonstrated the spatiotemporal properties of the [Ca^2+^]_i_ dynamics evoked by electrical stimulation in primary visual cortical slice preparations by means of Ca^2+^ imaging. The spatio-temporal distribution of the high [Ca^2+^]_i_ regions revealed the propagation properties of the action potentials in the primary visual cortical neuronal circuit. The evoked [Ca^2+^]_i_ transients in the cell somata are thought to be caused by the generation of action potentials [1], [2] and not by the evoked excitatory postsynaptic potentials in the absence of an action potential [3]. Indeed, when the action potential was blocked by TTX, no evoked [Ca^2+^]_i_ transients were observed in the case of 80 µA stimulation, and the [Ca^2+^]_i_ transients almost completely disappeared in the case of 200 µA stimulation (Figures 2, 4, and S3; see Results). Although the evoked [Ca^2+^]_i_ transients were greatly reduced when excitatory synaptic transmissions were blocked (Figures 2, 4, and S3), the time courses of the [Ca^2+^]_i_ transients at the stimulus position were almost identical (Figure S3). These results suggest that the evoked [Ca^2+^]_i_ transients were mainly due to the depolarization induced by the action potential. In the case of CNQX and AP5 administration, the some responses remained both at the stimulus position and at layer 2/3 (see Results). These remaining responses might include the Ca^2+^ release from intracellular Ca^2+^ stores via activation of the G-protein coupled receptors such as metabotropic glutamate receptors.

In individual neurons, the amplitude of the [Ca^2+^]_i_ transients in the burst activity vary with the firing number [1]–[3]. Ca^2+^ imaging potentially has a high spatial resolution of more than a single cell level [1], [2]. Indeed, some patchy structures that we observed may correspond to cell bodies, especially just above the stimulus site of layer 2/3 (Figures 2B and S2). This result suggests that specific neurons may be highly active in response to layer 4 stimulation. However, we are not able to conclude that specific neurons received strong synaptic inputs from layer 4. Electrical stimulation of a massive tissue, such as in a slice preparation, causes [Ca^2+^]_i_ transients within various cell depths, resulting in fluorescence changes of the Ca^2+^-indicator coming from every depth in the tissue. This prevents the detection of the location of the active cell somata. A laser scanning confocal microscope should be used to obtain single-cell spatial resolution rather than a conventional epifluorescent microscope. Moreover, the amplitudes of ΔF/F could differ based on the concentration of the Ca^2+^-sensitive dye loaded in the cell [3]. Due to this variation, binned regions were used for the quantitative analyses (Figures 3, 4, 6, S3, S4, and S6). Nonetheless, the high ΔF/F area likely corresponded well with the active area in our observations.

### The pathway of signal propagation

The high [Ca^2+^]_i_ region induced by layer 4 stimulation propagated mainly toward the dorsal direction (to layer 2/3) from the stimulus position, with restricted horizontal signal propagation (Figures 2, 3, and 4). We measured the onset of the [Ca^2+^]_i_ transients as the time when ΔF/F exceeded a threshold value (threshold ΔF/F = 0.5%). The onset of the [Ca^2+^]_i_ transients were slower at the layer 2/3 above the stimulus position compared with that at stimulus position. In the case of 80 µA stimuli, the average onset time was 19±4 ms at layer 2/3 (region a of Figure 3) and 8±1 ms at the stimulus position (region b of Figure 3) (n = 9 slices; *p*<0.05; paired *t*-test). When excitatory synaptic transmission was blocked by the application of CNQX and AP5, the amplitudes of the [Ca^2+^]_i_ transients around the stimulus position and at layer 2/3 were reduced, the response widths shrank, and anisotropic nature (dorsally elongation) of the high [Ca^2+^]_i_ region disappeared (Figures 2 and 4; see Results). These observations suggest that the evoked signal propagated from layer 4 to layer 2/3 mainly due to the excitatory synaptic transmission rather than the antidromic action potentials. The anisotropic signal propagation under the control condition is assumed to be due to the anisotropy of the functional (not physical) synaptic connections in the visual cortex (see next section).

### The function of inhibitory synaptic transmission

Intracortical connections are thought to run vertically, conveying information from layer 4 to the layers above and below [36]. Indeed, Hubel and Wiesel [37] demonstrated that neurons having similar orientation specificities aligned at right angle to the surface of the visual cortex. There are two possible explanations for this functional structure: 1) physical neuronal connections only run vertically in the visual cortical circuit; and 2) some inhibitory processes suppress horizontal signal propagation and form the functional column. The former assumption explains the vertical signal propagation from layer 4 under the control conditions as observed by Ca^2+^ imaging and indicates that the signal propagation properties may change little even if the inhibitory process transmission is blocked. Indeed, the signal evoked by layer 4 stimulation preferentially propagated vertically to layer 2/3 rather than horizontally (Figures 2, 3, and 4). This horizontal restriction of the signal propagation, however, disappeared when GABA_A_ receptors were blocked with bicuculline (Figures 5, 6, and S4). In addition, the largest [Ca^2+^]_i_ transients were observed in layer 2/3 when inhibitory synaptic transmission was blocked (Figures 5 and 6), and the time-to-peak values (>400 ms) were considerably longer than those under the control conditions (approximately 30 ms) (Figures 5, S4, and S5). [Ca^2+^]_i_ elevation is expected to be prolonged during burst activity [2], [3] which would explain such long time-to-peak values. Thus, postsynaptic neurons may receive excitatory synaptic inputs in a repetitive manner. Taken together, these results suggest that recurrent excitatory connections should exist [38]–[40], and inhibitory synaptic transmission suppresses horizontal signal propagation under normal conditions. Burkhalter [41] showed that the projection of a layer 2/3 neuron was widely spread in the horizontal direction, which provides support for the latter assumption. In fact, the inhibitory mechanisms contribute to the orientation selectivity [42]–[45]. Thus, inhibitory synapses are thought to play a critical role in the creation of functional sub-networks in the visual cortical circuit to form a columnar structure. Under such hyperactive conditions, however, G-protein coupled receptors such as metabotropic glutamate receptors may be activated. Therefore, the possible involvement of Ca^2+^ release from intracellular Ca^2+^ stores to prolong the [Ca^2+^]_i_ transients under bicuculline administration cannot be excluded completely.

To confine the propagation of excitatory signals, feed-forward inhibitions having a wide projection area should be required. If only feedback inhibitions exist, excitatory signal propagation could not be arrested. In the rodent primary visual cortex, both pyramidal-to-interneuron connections and interneuron-to-pyramidal connections exist [39], [40], [46]. The axon arbors of the interneurons are dense and widely spread, particularly in layer 2/3 [39]. In addition, there are a large number of interneurons in layer 2/3 as compared with the other cortical layers [32]. Thus, the feed-forward inhibitions, as well as feedback inhibition, to pyramidal cells are functionally suggested to exist, and these inhibitions may have a wide area of innervation, particularly in layer 2/3.

### Differences from previous studies using voltage-sensitive dyes

In many studies involving signal propagation in the visual cortex using voltage-sensitive dyes, the stimulation was applied to white matter [14]–[16], [18]–[20]. Because geniculocortical axons terminate mainly in layer 4 of the visual cortex [47]–[49], we applied the stimulation to layer 4 to reveal the visual signal propagation in the visual cortex.

There have been many studies on signal propagation in cortical slice preparations using voltage-sensitive dyes [14]–[20], [50], [51]. In these studies, the width of the horizontal signal propagation was >1 mm [14]–[16], [18]–[20], but here, the width of the horizontal signal propagation was restricted to approximately 200 µm. This difference may be explained by the difference in the signal source for the two types of dyes. The major signal source for voltage-sensitive dyes is thought to be a postsynaptic potential rather than an action potential [21]. In contrast, the major signal source of Ca^2+^-sensitive dye is thought to be an action potential [1] [2]. Thus, postsynaptic potentials below the threshold were detected within such a wide area. Indeed, the signal propagation to layer 2/3 from layer 4 almost disappeared when excitatory synaptic transmission was blocked in our study. In addition, studies that used voltage-sensitive dyes applied stimulation to white matter, with the evoked signal propagating from layer 6 to layer 1. In contrast, the evoked signal in our study propagated from layer 4 to layer 2/3. Because white matter also contains the output axons from the pyramidal cells of the visual cortex, both antidromic and orthodromic transmissions might occur during white matter stimulation. Yuste et al. [17] observed no significant changes in the laminar responses between stimulation under control conditions and stimulation under CNQX and AP5 administration and thus concluded that the signal of the voltage-sensitive dye also contained the ortho- and antidromic presynaptic signals. In our study, the signal propagation to layer 2/3 almost disappeared by blocking the excitatory synaptic transmission, suggesting that the Ca^2+^-imaging with layer 4 stimulation follows orthodromic action potential propagation in the visual cortical circuit.

In the somatosensory cortex, the horizontal extent of the signal propagation in layer 4 is restricted, even if inhibitory synaptic transmission is blocked [51], which is strikingly different from our results (Figures 4, 6, and S5). Thus, the architecture of the neuronal circuit in the somatosensory area may be different from that of the visual cortical area. Petersen and Sakmann [51] indicated that the time-to-peak of the signal of the voltage-sensitive dye slowed, and many action potentials were observed after application of bicuculline. These observations are in accordance with our own (Figures 5, S4, and S6), indicating that GABAergic interneurons may regulate the excitability of the recurrent network in both the somatosensory and visual cortexes.

The Ca^2+^ imaging technique has an advantage in the signal-to-noise ratio compared with voltage-sensitive dye imaging [1], [2]. This advantage permits quantitative analyses of the neuronal activities and is well suited for certain comparisons between animals (e.g., between wild type and transgenic animals). A comparison of results from low signal-to-noise ratio observations obtained from different animals is difficult. Many previous studies that used voltage-sensitive dyes involved rats or guinea pigs as the experimental animals [14]–[19], [51]. For the types of research that utilize transgenic animals, mice are widely used. Thus, the results herein should also provide fundamental data that can be applied to transgenic research.

## Materials and Methods

All experiments were approved by the Institutional Animal Care and Use Committee of the Graduate School of Engineering, Osaka University (permit number 17-6-0) and were conducted in accordance with the guidelines established by the Ministry of Education, Culture, Sports, Science and Technology, Japan.

### Slice preparation

Standard slice preparation protocols were used [30]. Briefly, postnatal day 14 (P14) to P28 C57BL/6J mice were anesthetized with halothane and decapitated. The cerebrum was rapidly isolated and placed in ice-cold normal artificial-cerebrospinal fluid (ACSF) bubbled with 95% O_2_–5% CO_2_. The composition of normal ACSF was as follows (in mM): 137 NaCl, 2.5 KCl, 0.58 NaH_2_PO_4_, 1.2 MgCl_2_, 2.5 CaCl_2_, 21 NaHCO_3_, and 10 glucose. Coronal slices of the visual cortex (thickness, 300 µm) were prepared using a vibratome tissue slicer (VT-1000S; Leica Microsystems, Nussloch, Germany). The slices were incubated at room temperature in a submerged chamber containing gassed ACSF for at least 60 min prior to the experiments.

### Ca^2+^ imaging and stimulation

[Ca^2+^]_i_ in the slices was measured using the membrane-permeant acetoxymethyl (AM) ester of Oregon Green 488 BAPTA-1 (OGB1-AM; Invitrogen, Carlsbad, CA) dissolved in dimethylsulfoxide (DMSO; Dojindo Laboratories, Kumamoto, Japan). The visual cortical slices were placed in a small plastic Petri dish containing 100 µl ACSF with 10 µM OGB1-AM and 0.02% Cremophor EL (Sigma, St. Louis, MO). The dish was incubated at 35°C for 30 min in a small chamber, which was humidified and continuously aerated with 95% O_2_–5% CO_2_, and then washed with 100 µl ACSF at 35°C for 15 min. OGB1-loaded slices were transferred to a continuously superfused (2–2.5 ml/min) chamber on the stage of a BX51WI epifluorescent upright microscope (Olympus, Tokyo, Japan). The excitation light source (770 U with a 150 W Xenon arc lamp; Opti Quip, Highland Mills, NY) was coupled to the epifluorescent port of the microscope equipped with the filter cube (XF100-2; Omega Optical, Brattleboro, VT) for excitation and emission. The fluorescence signals were imaged with a 20×, NA 0.95 water-immersion objective (Olympus) at 29–31°C and were captured using a cooled-CCD NeuroCCDsm256 camera (256×256 pixels; Redshirt Imaging, Decatur, GA) at a frame rate of 100 Hz (100 fps). The actual imaging area was 640×640 µm. The [Ca^2+^]_i_ transients were evoked by stimulation with a 200 µs, 40 to 240 µA biphasic current from a glass microelectrode (tip diameter, 10–20 µm) placed in layer 4 of the visual cortical slice. The stimulation with each current under each condition was repeated 3–10 times, and the responses were averaged. Because a current stimulator generally has internal impedance, the actual amount of the stimulus current is likely different from the command current pulse. To confirm this issue, we measured the actual stimulus current. The current wave forms, when various intensities of 100 µs negative and 100 µs positive square command pulses were applied to the stimulus electrode in saline, are shown in Figure S1. The stimulation currents were recorded by the voltage drop between a 10 kΩ resistor, which was inserted in series in the stimulus circuit. The current amplitudes were small compared to the command amplitudes, and the waveforms were not square. The amplitudes, however, were almost proportional to the command amplitudes. Therefore, the amplitudes of the responses evoked by the stimulation were normalized for quantitative analysis (in Figures 4 and 6), and the stimulus current amplitudes were indicated by the command current amplitudes.

### Data analysis

Images were recorded using NeuroPlex software (Redshirt Imaging) and processed using custom-made MATLAB (MathWorks, Natick, MA) programs. For noise reduction, the captured fluorescence images were spatially filtered with a 5×5 Gaussian Kernel, and the time series of the images were temporally filtered with a Hanning filter (window width, 3 frames). The relative changes in [Ca^2+^]_i_ were quantified as ΔF/F, where F indicates the average fluorescence intensity before stimulation, and ΔF is the change in fluorescence intensity from F. For quantitative data analyses, the 256×256 fluorescence images were binned by 16×16 pixels (actual bin size, 40×40 µm) followed by an application of the Hanning filter to the time series of the binned images and the calculation of ΔF/F (Figures 3, 4, 6, S3, S4, and S5).

### Layer determination

During the experiment, the cortical layer boundaries were identified from the infrared differential interference contrast (IR-DIC) images based on cell shape, size, and density. To confirm the layer organization and stimulus position after the experiment, the position of the stimulus electrode tip was marked by UV-laser irradiation (AVIA Ultra 355-350; Coherent, Santa Clara, CA). Some slices were fixed with 4% paraformaldehyde in phosphate-buffered saline (PBS), followed by staining with cresyl violet (Nissl stain). The stimulus position and layer organization were confirmed using a microscope (BX51WI). The approximate thicknesses of layer 2/3, layer 4, and layer 5 were 250–350 µm, 100 µm, and 300–400 µm, respectively. These thicknesses were compatible with other estimates from the literature [31], [32].

### Data representation and statistical analysis

The left-hand sides of all 2D images or the 2D time courses in the Figures correspond to the lateral side of the cortical slice. To omit fluctuation of the baseline in the pseudocolor images, any ΔF/F values less than three times the standard deviation obtained from the ΔF/F values of 20 frames before stimulation were treated as zero.

All data are presented as means ± SEM (standard error of the mean) unless stated otherwise. Differences were considered significant at *p*<0.05 by statistical testing.

### Drugs

All drugs were applied by perfusion. Tetrodotoxin (TTX; 1 µM; Alomone Labs, Jerusalem, Israel) was used to block action potentials. 6-cyano-7-nitroquinoxaline-2,3-dione (CNQX; 10 µM; Tocris, Bristol, United Kingdom) and DL-2-amino-5-phosphonovaleric acid (AP5; 50 µM; Tocris) were used to block excitatory synaptic transmission. The GABA_A_ receptor antagonists (+)-bicuculline (Bicuculline; Tocris) and picrotoxin (Tocris) were used to block inhibitory synaptic transmission.

## Supporting Information

Figure S1 Waveforms of actual stimulus currents. The currents were measured by a digital storage oscilloscope. Command current amplitudes are indicated in the graph legends.
(0.40 MB PDF)

Figure S2 Pseudocolor image of the peak ΔF/F (left panel) and IR-DIC (right panel) image of the same slice shown in Figures 2A, 2B and 3. The black oval regions mark the same position in both images. Scale bar  =  100 μm.
(0.55 MB PDF)

Figure S3 Time courses of the [Ca^2+^]_i_ transients evoked by layer 4 stimulation. The evoked [Ca^2+^]_i_ transients obtained from the 16 × 16-pixel binned area under the control condition (black line), under the condition of 10 μM CNQX + 50 μM AP5 administration (red line), and under the condition of 1 μM TTX administration (blue line) in the same slice shown in Figures 2A, 2B and 3. The illustrations are the same as those in Figure 3A. The stimulus intensity was 80 μA. The stimulus region is indicated by the red dashed-line box, and the center-to-center distance of each panel was 80 μm. Scale bar  =  100 ms; ΔF/F  =  6%.
(0.40 MB PDF)

Figure S4 Time courses of the [Ca^2+^]_i_ transients evoked by layer 4 stimulation under bicuculline administration. The [Ca^2+^]_i_ transients evoked by the application of an 80 μA stimulus to layer 4 obtained from the 16 × 16-pixel binned area under the control condition (black line), under the condition of 2 μM bicuculline administration (red line), and under the condition of 5 μM bicuculline administration (blue line) in the same slice shown in Figures 5A, B and C. The illustrations are the same as those in Figure S3. Scale bar  =  500 ms; ΔF/F  =  5%.
(0.49 MB PDF)

Figure S5 Time-to-peaks of the [Ca^2+^]_i_ transients under the control condition and under the administration of 5 μM bicuculline. (A) The values of time-to-peak were obtained from the 16 × 16-pixel binned data in the three red regions (vertical, L2/3 horizontal, and L4 horizontal) on the pseudocolor [Ca^2+^]_i_ transient images. The vertical line is drawn perpendicular to the cortical layers and passes through the stimulus position. The L2/3 horizontal line is drawn along layer 2/3, and its vertical distance from the stimulus position is 200 μm. The L4 horizontal line is drawn along layer 4 and passes through the stimulus position. Scale bar  =  100 μm. The distributions of the time-to-peaks (B) of the [Ca^2+^]_i_ transients under the control condition (filled circles) and under the administration of 5 μM bicuculline (open circles) along with the vertical, L2/3 horizontal, and L4 horizontal lines. In the vertical panel, the distance indicates the displacement from the stimulus position, with negative corresponding to the dorsal direction and positive to the ventral direction. In the L2/3 horizontal and L4 horizontal line panels, the distance indicates the horizontal displacement from the stimulus position, with negative corresponding to the lateral direction and positive to the medial direction. Error bars are represented as SEM.
(0.44 MB PDF)

Figure S6 Blockade of inhibitory synaptic transmission by picrotoxin also enhanced the propagation of the evoked signal. Time-lapse pseudocolor images of the [Ca^2+^]_i_ transients evoked by the application of the 120 μA stimulus in layer 4 under the control condition (A) and under the condition of 10 μM picrotoxin administration (B). The illustrations are the same as those in Figures 2 and 5. Scale bar  =  100 μm.
(1.25 MB PDF)
