# Supplementary figures and images for: Spatiotemporal Properties of the Action Potential Propagation in the Mouse Visual Cortical Slice Analyzed by Calcium Imaging
Source: PLoS One. 2010 Oct 29;5(10):e13738. doi: 10.1371/journal.pone.0013738 (PMC2966408; doi:10.1371/journal.pone.0013738)

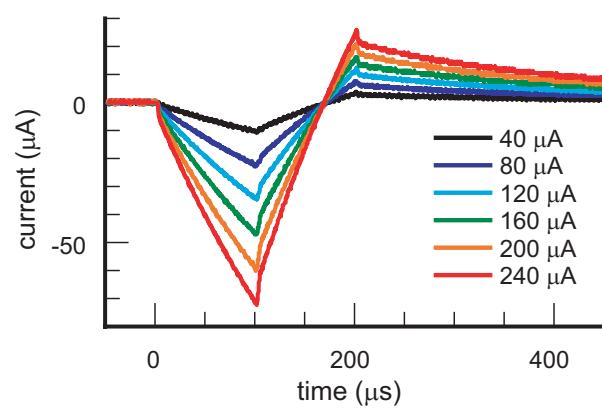

Supplement: Figure S1 — Waveforms of actual stimulus currents. The currents were measured by a digital storage oscilloscope. Command current amplitudes are indicated in the graph legends. (0.40 MB PDF) [file pone.0013738.s001.pdf]

peak  $\Delta F/F$

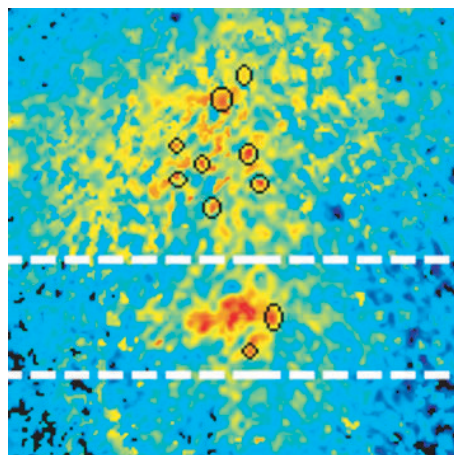

IR-DIC

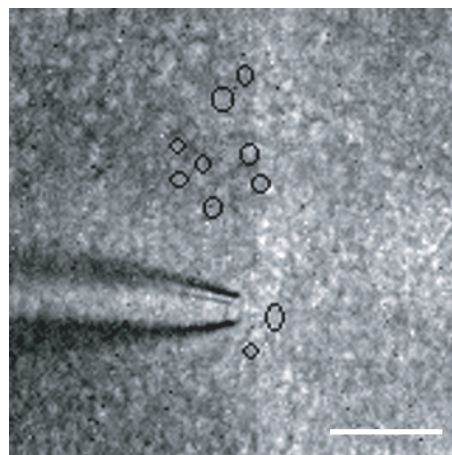

Supplement: Figure S2 — Pseudocolor image of the peak ΔF/F (left panel) and IR-DIC (right panel) image of the same slice shown in Figures 2A, 2B and 3. The black oval regions mark the same position in both images. Scale bar = 100 μm. (0.55 MB PDF) [file pone.0013738.s002.pdf]

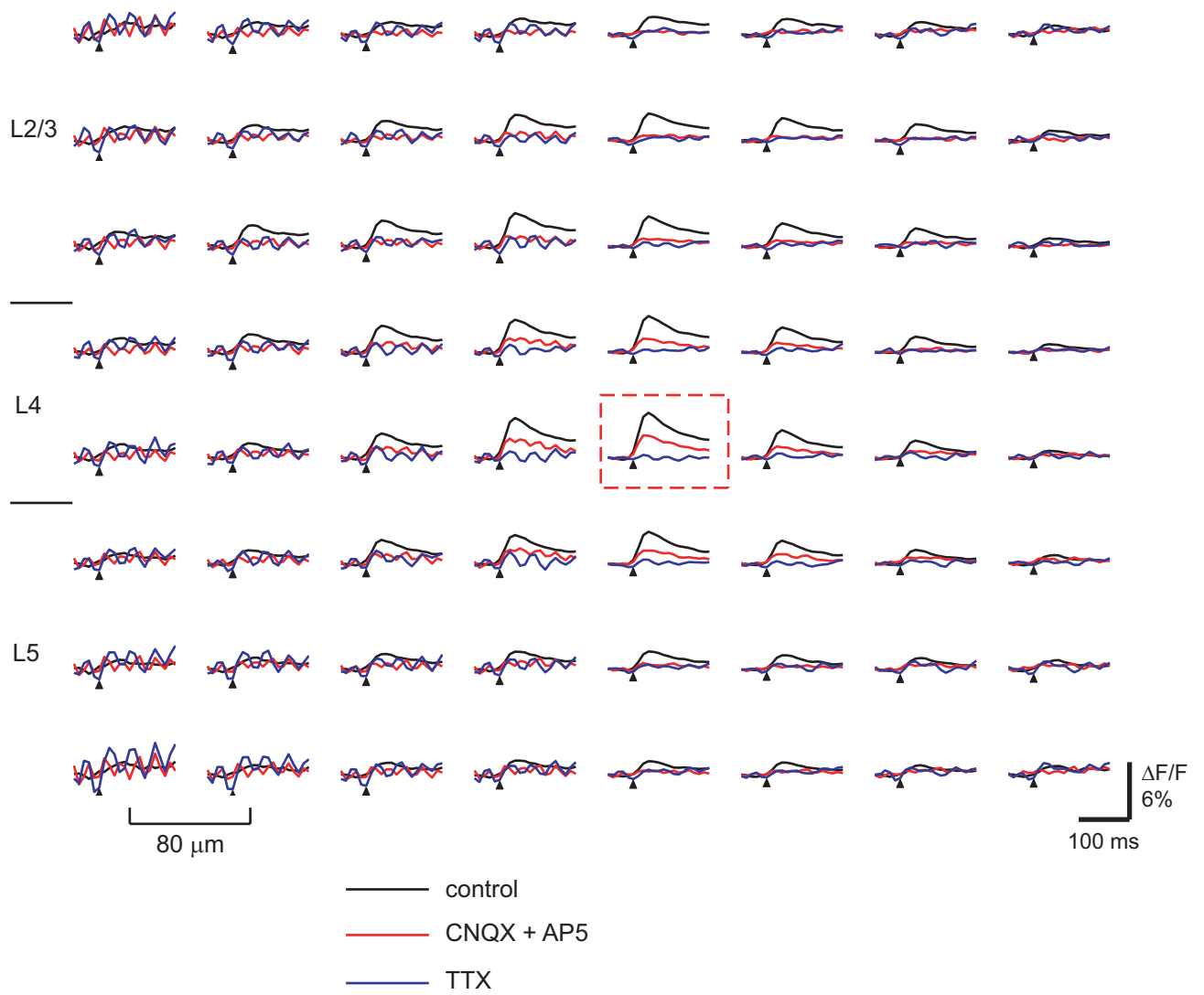

Supplement: Figure S3 — Time courses of the [Ca2+]i transients evoked by layer 4 stimulation. The evoked [Ca2+]i transients obtained from the 16 × 16-pixel binned area under the control condition (black line), under the condition of 10 μM CNQX + 50 μM AP5 administration (red line), and under the condition of 1 μM TTX administration (blue line) in the same slice shown in Figures 2A, 2B and 3. The illustrations are the same as those in Figure 3A. The stimulus intensity was 80 μA. The stimulus region is indicated by the red dashed-line box, and the center-to-center distance of each panel was 80 μm. Scale bar = 100 ms; ΔF/F = 6%. (0.40 MB PDF) [file pone.0013738.s003.pdf]

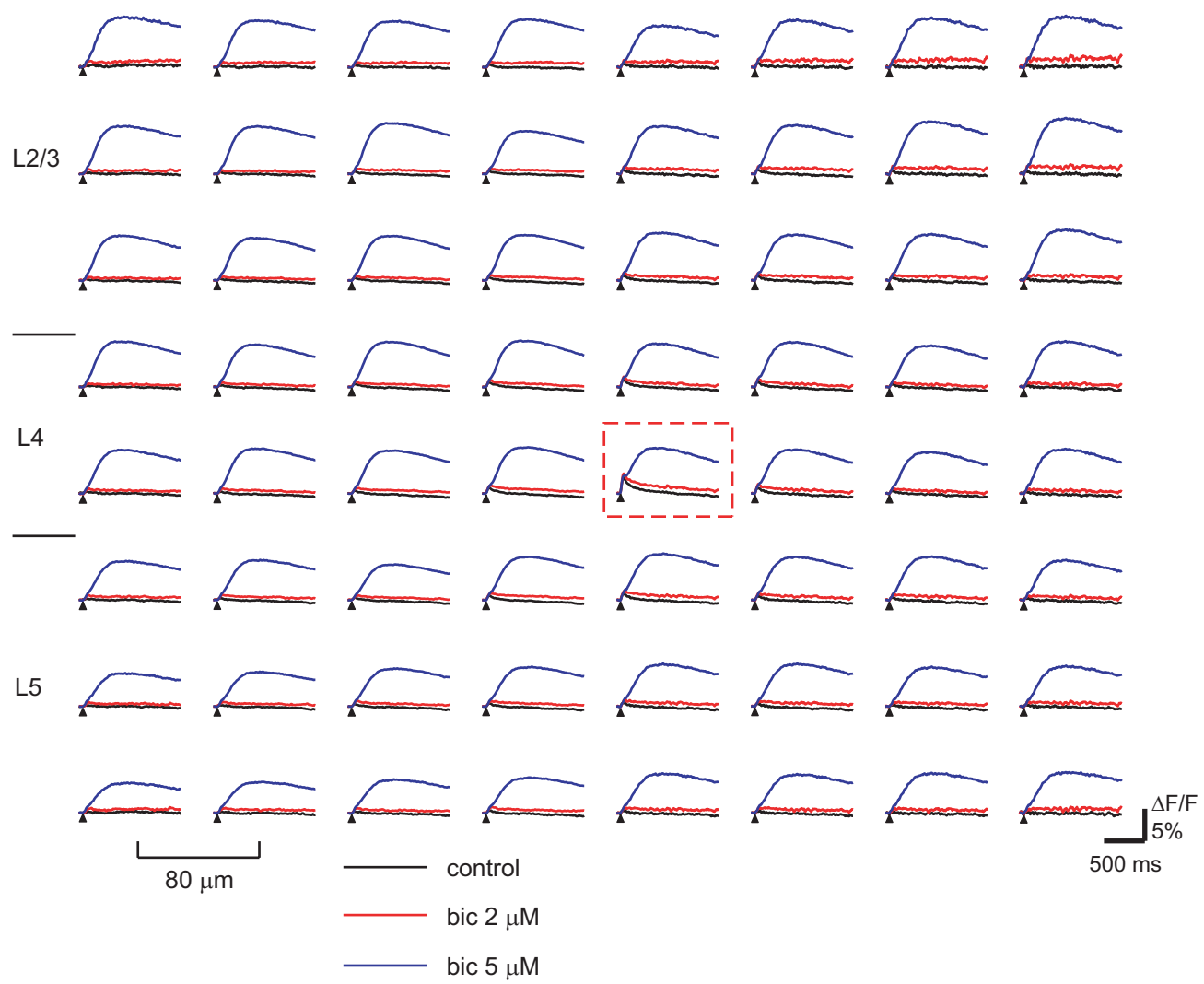

Supplement: Figure S4 — Time courses of the [Ca2+]i transients evoked by layer 4 stimulation under bicuculline administration. The [Ca2+]i transients evoked by the application of an 80 μA stimulus to layer 4 obtained from the 16 × 16-pixel binned area under the control condition (black line), under the condition of 2 μM bicuculline administration (red line), and under the condition of 5 μM bicuculline administration (blue line) in the same slice shown in Figures 5A, B and C. The illustrations are the same as those in Figure S3. Scale bar = 500 ms; ΔF/F = 5%. (0.49 MB PDF) [file pone.0013738.s004.pdf]

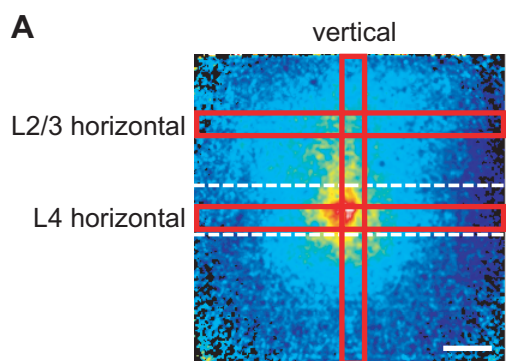

**B** time-to-peak

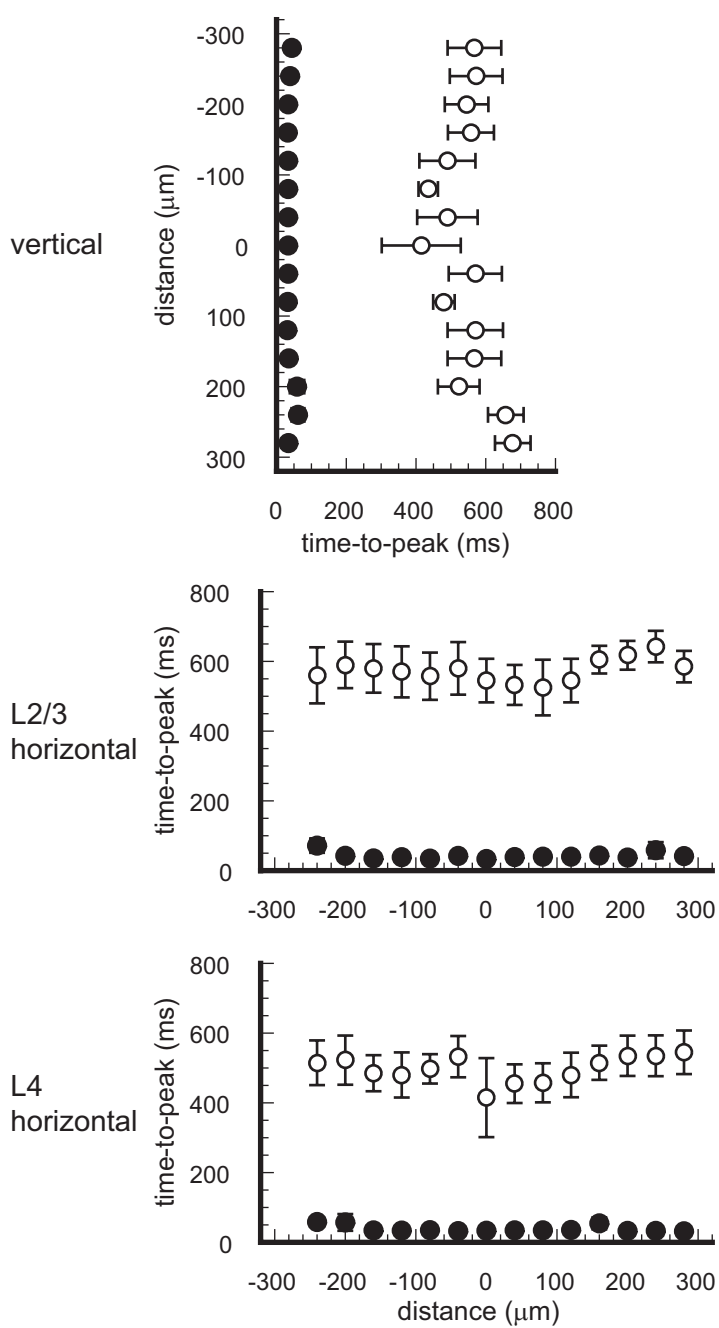

Supplement: Figure S5 — Time-to-peaks of the [Ca2+]i transients under the control condition and under the administration of 5 μM bicuculline. (A) The values of time-to-peak were obtained from the 16 × 16-pixel binned data in the three red regions (vertical, L2/3 horizontal, and L4 horizontal) on the pseudocolor [Ca2+]i transient images. The vertical line is drawn perpendicular to the cortical layers and passes through the stimulus position. The L2/3 horizontal line is drawn along layer 2/3, and its vertical distance from the stimulus position is 200 μm. The L4 horizontal line is drawn along layer 4 and passes through the stimulus position. Scale bar = 100 μm. The distributions of the time-to-peaks (B) of the [Ca2+]i transients under the control condition (filled circles) and under the administration of 5 μM bicuculline (open circles) along with the vertical, L2/3 horizontal, and L4 horizontal lines. In the vertical panel, the distance indicates the displacement from the stimulus position, with negative corresponding to the dorsal direction and positive to the ventral direction. In the L2/3 horizontal and L4 horizontal line panels, the distance indicates the horizontal displacement from the stimulus position, with negative corresponding to the lateral direction and positive to the medial direction. Error bars are represented as SEM. (0.44 MB PDF) [file pone.0013738.s005.pdf]

**A (control)**

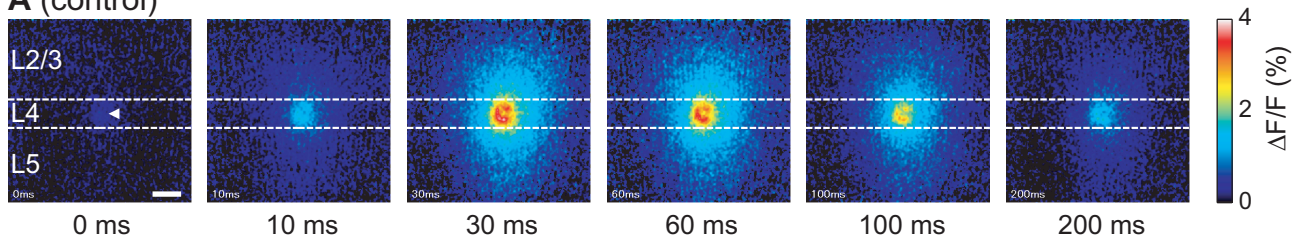

**B (picrotoxin 10  $\mu$ M)**

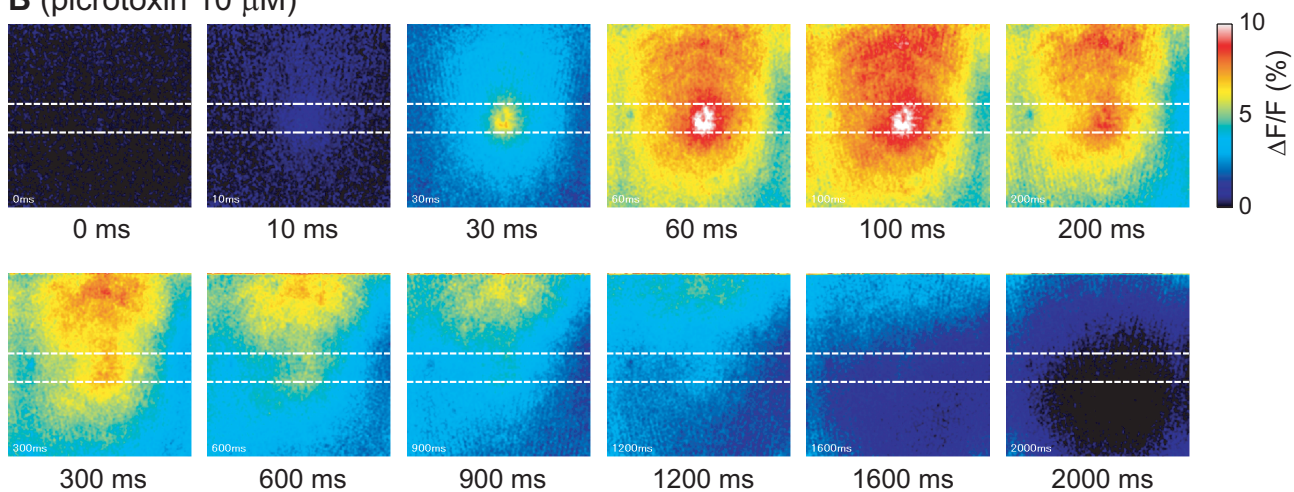

Supplement: Figure S6 — Blockade of inhibitory synaptic transmission by picrotoxin also enhanced the propagation of the evoked signal. Time-lapse pseudocolor images of the [Ca2+]i transients evoked by the application of the 120 μA stimulus in layer 4 under the control condition (A) and under the condition of 10 μM picrotoxin administration (B). The illustrations are the same as those in Figures 2 and 5. Scale bar = 100 μm. (1.25 MB PDF) [file pone.0013738.s006.pdf]
